# Incidental giant subserosal lipoma of the transverse colon with fat necrosis and abscess formation discovered during emergency laparotomy for blunt abdominal trauma: a case report

**DOI:** 10.1093/jscr/rjag913

**Published:** 2026-10-10

**Authors:** Mahmoud Mojahed, Mahmoud Al-Zaro, Raneen Shaher Alkaraki, Rima Awawdeh, Mutasem Sayyed Ahmad, Shahd Al-Qasrawi, Sulaiman NajiFares Fakhouri

**Affiliations:** Department of Internal Medicine, Al-Ahli Hospital, Be'er Almahjar area (Farsh Al Hawa), Hebron P700, Palestine; Faculty of Medicine, Al-Quds University, Abu Dis, Jerusalem P100, Palestine; Faculty of Medicine and Health Sciences, Palestine Polytechnic University, Wadi Hariya Campus, Hebron P700, Palestine; Faculty of Medicine and Health Sciences, Palestine Polytechnic University, Wadi Hariya Campus, Hebron P700, Palestine; Department of General Surgery, Hebron Governmental Hospital (Alia), King Faisal Street, Hebron P700, Palestine; Department of General Surgery, Hebron Governmental Hospital (Alia), King Faisal Street, Hebron P700, Palestine; Department of General Surgery, Hebron Governmental Hospital (Alia), King Faisal Street, Hebron P700, Palestine

**Keywords:** colonic lipoma, subserosal lipoma, blunt abdominal trauma, fat necrosis, emergency laparotomy, case report

## Abstract

Giant subserosal colonic lipomas are rare, benign tumors that are typically asymptomatic. However, they can mimic malignancy or become complicated by inflammation and necrosis. We present a 59-year-old male who underwent emergency laparotomy for a traumatic splenic rupture following blunt abdominal trauma. During surgery, a 12 × 10 cm incidental mass in the transverse colon was discovered, mimicking a malignant tumor. Due to gross purulent intra-abdominal contamination from an intratumoral abscess and patient instability, a segmental colonic resection with an end colostomy was performed alongside the splenectomy. Histopathology confirmed a giant subserosal lipoma complicated by fat necrosis and abscess formation. This case highlights a highly unusual presentation of an inflamed colonic lipoma discovered incidentally during trauma surgery. It underscores the diagnostic difficulty of benign tumors in acute settings and supports definitive surgical resection when malignancy cannot be excluded.

## Introduction

Colonic lipomas are the second most prevalent benign tumors of the colon, following adenomatous polyps, with an incidence between 0.035% and 4.4% [1]. These mesenchymal neoplasms are composed of mature adipose tissue and are typically found in the submucosa. Most are small, asymptomatic, and found incidentally during a colonoscopy. However, larger tumors can cause bleeding, obstruction, or intussusception [2].

Subserosal lipomas, originating from the appendices epiploicae or subserosal fat, are considerably less common than submucosal lipomas. Due to their anatomic location, they rarely block the lumen, but they can complicate diagnosis by compressing nearby organs or mimicking cancerous masses. Inflammation, infarction, or fat necrosis within a lipoma is uncommon and usually associated with torsion or ischemia [3]. Moreover, it is rarely reported for a lipoma to have an intratumoral abscess.

In blunt abdominal trauma, the surgical priority is identifying and controlling hemorrhage. However, incidental findings during exploratory laparotomy make it more challenging to make decisions during the procedure, particularly when a mass mimics a malignancy with associated lymphadenopathy. We present a case of a 59-year-old male who underwent emergency laparotomy for a traumatic splenic rupture, revealing a giant subserosal lipoma complicated by fat necrosis and abscess formation. This highlights the importance of thorough exploration and the need to consider benign pathology even in the presence of alarming macroscopic features.

## Case report

A 59-year-old male admitted following a motor vehicle accident resulting in blunt abdominal trauma. He presented with severe abdominal, chest, and back pain. Initially, the patient was hemodynamically stable. A focused assessment with sonography for trauma examination demonstrated perihepatic and pelvic free fluid. He subsequently developed hypotension, necessitating urgent surgical intervention. The patient had no major medical comorbidities. His past surgical history included a laparoscopic cholecystectomy.

An emergency exploratory laparotomy was performed through a midline incision extending from the xiphoid to the suprapubic region. Upon entering the abdomen, purulent fluid was encountered and drained (Fig. 1), alongside significant intraperitoneal blood and pelvic clots. Exploration revealed a traumatic splenic injury with active bleeding; the splenic pedicle and splenic artery were identified, ligated, and a splenectomy was completed following division of the gastrosplenic ligament. The liver was intact and packed for hemostasis. Further bowel exploration demonstrated a palpable mass involving the transverse colon with enlarged mesenteric lymph nodes. The mesentery was opened proximally and distally, intestinal clamps were applied, and a segmental colonic resection was performed. The distal colonic stump was closed, and a right flank end colostomy was created. The abdomen was copiously irrigated and closed in layers with surgical drains placed. The patient received intraoperative blood products and was transferred to recovery in stable condition.

**Figure 1:**
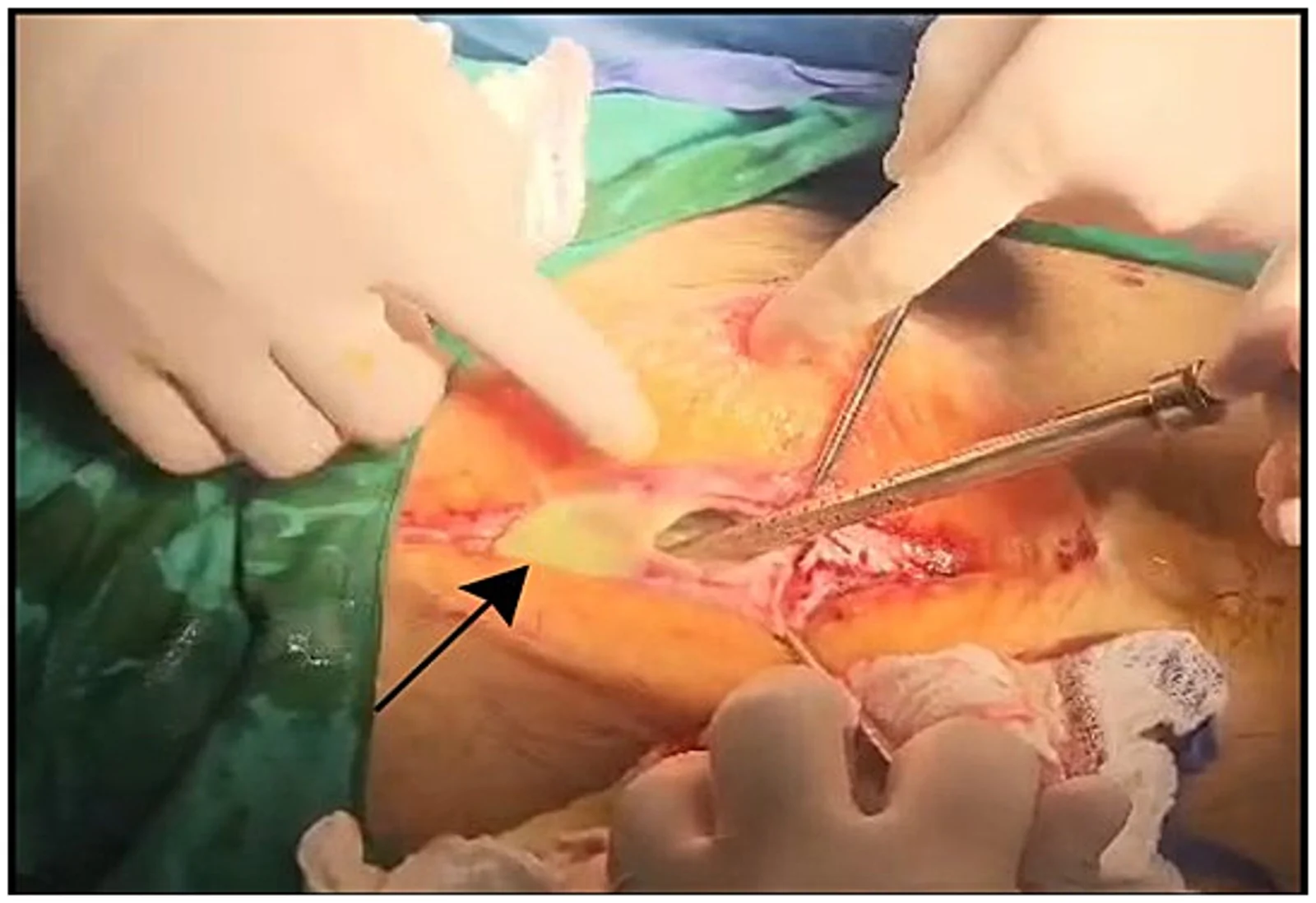
Intraoperative photograph revealing a collection of purulent exudate (arrow) upon entering the abdominal cavity.

Histopathological examination of the resected colonic specimen revealed a large subserosal lipoma measuring 12 × 10 cm (Fig. 2). The mass predominantly comprised mature adipocytes lacking nuclear atypia, but was associated with active and chronic inflammation, fat necrosis, and abscess formation. Resection margins were free of malignancy, and twelve retrieved lymph nodes showed reactive changes only. The spleen demonstrated red pulp congestion without malignancy.

**Figure 2:**
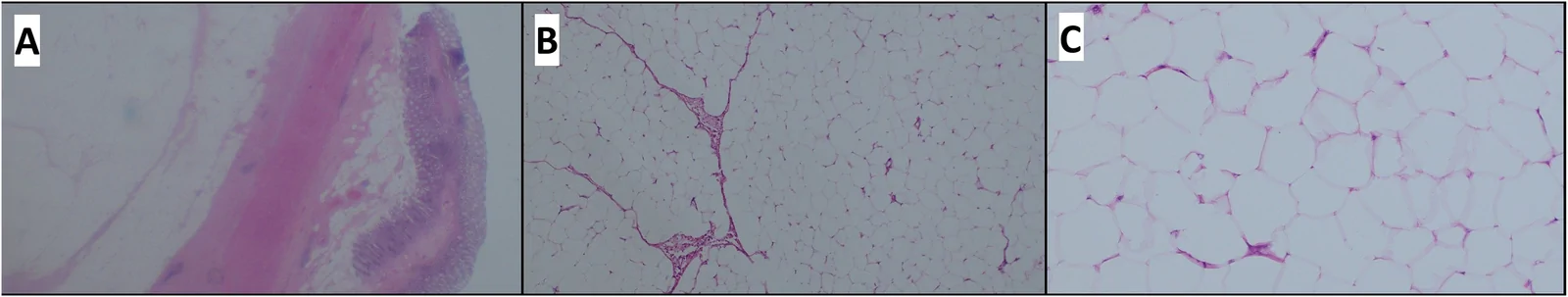
Histopathological examination of the resected colonic specimen. (A) Whole mount view (hematoxylin and eosin stain) demonstrating a broad subserosal mass composed of adipose tissue adjacent to the colonic mucosa and muscularis. (B) Low-power (4×) and (C) medium-power (10×) views showing mature adipocytes lacking nuclear atypia, confirming the diagnosis of a benign lipoma.

Postoperatively, the patient’s course was complicated by a superficial surgical site infection and seroma at the midline laparotomy incision, which were managed conservatively with wound care and targeted antibiotics. He also developed reactive thrombocytosis requiring antiplatelet therapy. He showed gradual clinical improvement, tolerated oral intake, and regained mobility. He was discharged in stable condition with appropriate medical therapy, wound care instructions, follow-up arrangements, and a plan to receive post-splenectomy vaccinations.

## Discussion

Colonic lipomas are uncommon benign mesenchymal tumors composed of well-differentiated adipose tissue. They most commonly arise in the ascending colon, followed by the sigmoid and descending colon, whereas involvement of the transverse colon is rare. The majority originate from the submucosal layer; lipomas arising from the subserosal layer are distinctly uncommon and infrequently reported [4].

Most colonic lipomas are small and asymptomatic and are often identified incidentally during imaging, surgery, or autopsy. Lesions larger than 4 cm are classified as giant lipomas [5], and tumors exceeding 2 cm present with symptoms in nearly 75% of cases [1], Additionally, if development is directed toward the luminal side, symptoms manifest early, whereas if growth is directed toward the serosal side, presentation may be delayed. Abnormal bowel habits, diarrhea, gastrointestinal bleeding, abdominal pain, and episodes of intussusception or intestinal obstruction are the most typical signs of colonic lipoma [6].

In our case, a 12 × 10 cm subserosal lipoma was incidentally discovered during emergency laparotomy for blunt abdominal trauma. The patient’s acute presentation was due to traumatic splenic injury and hemoperitoneum, which required urgent surgical intervention. The presence of purulent fluid, fat necrosis, and abscess formation around the colonic lesion suggests chronic, subclinical inflammation. The blunt abdominal trauma likely disrupted this pre-existing condition, leading to peritoneal contamination and making the lesion noticeable. The connection between trauma and an inflamed colonic lipoma is rarely reported.

Preoperative diagnosis of colonic lipomas is often difficult, especially in emergency trauma cases where imaging focuses on life-threatening injuries. Intraoperatively, giant lipomas can mimic malignancy due to their size, firm consistency, inflammation, and regional lymphadenopathy [7].

Management of colonic lipomas depends on the size, symptoms, and clinical presentation. Small asymptomatic lipomas can be observed or removed endoscopically, while large or complicated lipomas require surgical resection [8, 9]. In an emergency setting with contamination, inflammation, or suspicion of malignancy, the decision is to do a segmental colonic resection. This resection with end colostomy in our patient was due to the abscess formation, intra-abdominal contamination, and the need to maintain the patient’s safety during damage-control surgery [8, 10].

This case illustrates a rare incidental finding of a giant inflamed colonic lipoma discovered during trauma surgery. It underscores the diagnostic difficulty of benign colonic tumors in acute settings and supports definitive surgical resection when malignancy cannot be excluded. Reporting such cases adds to the limited literature and may assist surgeons when unexpected colonic masses are encountered during trauma laparotomy.

## Data Availability

All data underlying the results are available as part of the article and no additional source data are required.

## References

[1] Jiang L, Jiang LS, Li FY et al. Giant submucosal lipoma located in the descending colon: a case report and review of the literature. World J Gastroenterol 2007;13:5664–7. 10.3748/wjg.v13.i42.5664 PMC4172750 17948945

[2] Janevska V, Spasevska L, Dukova B et al. Intestinal submucosal lipomas. Maced J Med Sci 2012;5:49–54. 10.3889/MJMS.1857-5773.2012.0199

[3] Zemenfes D, Semegnew Z. Subserosal sigmoid lipoma: a case report and review of the literature. Ethiop Med J 2016;54:87–9. 27476229

[4] Nallamothu G, Adler DG. Large colonic lipomas. Gastroenterol Hepatol (N Y) 2011;7:490–2. PMC3264900 22298986

[5] Paškauskas S, Latkauskas T, Valeikaitė G et al. Colonic intussusception caused by colonic lipoma: a case report. Medicina (Kaunas) 2010;46:477–81. 10.3390/medicina46070069 20966621

[6] Castro EB, Stearns MW. Lipoma of the large intestine: a review of 45 cases. Dis Colon Rectum 1972;15:441–4. 10.1007/BF02642650 4645612

[7] Bahadursingh AM, Robbins PL, Longo WE. Giant submucosal sigmoid colon lipoma. Am J Surg 2003;186:81–2. 10.1016/S0002-9610(03)00111-9 12842756

[8] Fernandez MJ, Davis RP, Nora PF. Gastrointestinal lipomas. Arch Surg 1983;118:1081–3. 10.1001/archsurg.1983.01390090065015 6615219

[9] Kim CY, Bandres D, Tio TL et al. Endoscopic removal of large colonic lipomas. Gastrointest Endosc 2002;55:929–31. 10.1067/mge.2002.124098 12024158

[10] Cirocchi R, Corsi A, Castellani E et al. Case series of non-operative management vs. operative management of splenic injury after blunt trauma. Ulus Travma Acil Cerrahi Derg 2014;20:91–6. 10.5505/tjtes.2014.99442 24740333

